# Cohnheim and Virchows Views of the Origin of Pus-Corpuscles

**Published:** 1868-08

**Authors:** 


### CORRESPONDENCE.

### ARTICLE V.

### *Cohnheim and Virchows Views of the Origin of Pus-Corpuscles.*

ENGLISH REPORTS OF THE SAME.

I beg to direct your attention to a case of propagation of an egregious error, embodied in the Periscopic Department, * of one of your cotemporaries. In the account there given of M. Cohnheim's interpretation of certain phenomena of cell gathering, induced by him, during experimentation upon the cornea and mesentery, we are told, that he has given a death blow to the theory which refers pus-corpuscles to the propagation of cells of connective tissue, and that he "has demonstrated the identity of white blood corpuscles or leucocytes, (Robin,) and pus-cells." However, the first particular may be, it is abundantly plain, that the representation of this claim for the doctor, is exceedingly puerile. This

* Dental Cosmos, "Periscope," Page 107, Vol. 10, No. 2, Art. "Identity of Pus and White Blood Corpuscles."

is doubtless the fault of the writer of the account in question. For whenever and wherever the English speak about Virchows cell doctrines the "old serpent" comes out with true "Bull" vigor. In fact, however the writer on points of Histology, for their current publications, especially always, those of the *Bri.* and *For. Med. Chir. Review and the Medical Times & Gazette*, are invariably "stock" writers, who possess none of the knowledge gained in habitual examination of tissues, comprising such elements as those in question. Hence in their destitution of this only real, qualifying knowledge, they invariably confound facts widely discriminated as totally unlike by habitual histologists. Hence in this case, the egregious blunder of identifying the contractile properties of pus-corpuscles and white blood corpuscles. When in fact no such identity of cell activity, or consumated change of dimensions of these two sets of bodies, has ever been reported as seen by any competent observer.

As to the demonstration of their identity, this has been reported as *un fait accompli*, so often in the same manner as now of Dr. Cohnheim, that one familiar with the fact, wonders that the Dr. should take the trouble, (or his reporter for him,) of again repeating this puerile claim. Always the wonder is repeated, that men for the sake of the little notoriety of print, of some claim to discovery, should assert along with their claim, what to every histologist, instantly reveals the baselessness of the claim itself, by carrying with their assertions the proof of its impossibility. So with M. Cohnheim's discoveries, the "pus-corpuscles," which he claims are actually white cells which have emigrated from the blood stream," have left their very much *smaller* brethren—the red-corpuscles—*without means of egress by the same route ;* have in fact come out, by means which keep the lesser and far more numerous bodies in.

Shall we not rather say, with all our knowledge to verify our statement, that these "white cells," seen by M. Cohnheim, came from canals or vessels, not the "*blood stream*," —from which they could not by any possibility find egress without an inundation of the tissues with other blood corpuscles, and substance—which carry only white corpuscles and fluid, namely, the lymph canals? So when we are told that M. Cohnheim, "observed step by step the emigration of white corpuscles through the walls of veins," we may well ask whether the statement is not one made to show how far the force of derision can go. But all this, is very little, as compared with our astonishment, when we come to read of the reported identity, discovered by the same observer of the pus-corpuscles, of the white blood corpuscles, and of the "contractile corpuscles" of Recklinghausen. As we had frequent sights of these "corpuscles," discovered by this able observer, in the year in which we studied side by side with him in Berlin, in —63, nothing could have more amazed us than to be told by the English report of M. Cohnheim's claim, of the identity between these "contractile corpuscles," and those of M. Cohnheim. In this confusion we find the most complete and demonstrative proof of the entire destitution of histological knowlege of the *actual* kind, of the writer of this report. The alleged identity in fact, is the utmost, or rather most total, unlikeness; for the two bodies in question resemble each other in *no particular*, not even the wholly *accidental* one of size. It is wholly within the bounds of the strictest scientific truth to say, that, than these two elements, nothing can be conceived more totally unlike.

The contractile corpuscles in question, are minute bodies, of apparently hyaline plastic substance. They have a clear wall like outline, and show the most singularly rapid changes of shape, which is always more or less irregular or completely stellate, by darting out a *part* of their otherwise rounded surface, into the form of a process or *projection*, or several of them in succession. Anything of any shape, more unlike *any* of the morphological characters of the pus-cells or white blood corpuscles, and their slight changes of posture or retractility, hardly, even in the *occasional instances where it really* exists, to be measured at all by sight, or appreciable to sense—cannot be mentioned. In fact, as I pointed out at the time to their discover, the socalled contractile action, is exactly speaking, *ex*-tensile, the apparent *re*-traction being in fact, induced by a new projection, of another part, thus inducing displacement interior-ward, of the part previously projected.

But the whole of the error we notice of the account in question, is not shown in the alleged identity of these totally unlike bodies, but is further shown, in the part of the report which first specifically states, Recklinghausen's interpretation of the morphological character of these truly contractile bodies, namely " their being formed from the first connective tissue corpuscles," and the subseqent contradiction of this statement, by affirming that Recklinghausen "*recognized* their morphological identity with pus-cells, lymph, and white blood corpuscles." Recklinghausen never stultified himself in this amazingly stupid self contradiction. The former alone, was his interpretation. But this contradiction of Recklinghausen's views, in which he is represented as stating two conclusions of the origin of these bodies, one entirely antagonistic to the other, is better, than another contradiction of M. Cohnheim's English reporter. The latter says Recklinghausen " alluded to the *probability* of their being formed from connective tissue corpuscles, and then assigns to him also the statement that " he was *acquainted* with the increase and accumulation of these wandering elements, &c., &c."

And so also, with the same obvious insensibility to discrimination, the same writer, quotes an experiment of Recklinghausen as in accord with, and in confirmation of M. Cohnheim's reported discoveries, which is in fact decidedly adverse to, and opposed to his claim, namely, the experiment involving the motile action of lymph corpuscles in lymph-sacs, and thus distinctively *precluding* any even the least participation in the phenomena induced, of M. Cohnheim's pathological elements from the *blood stream*, from artificialy or inflamed territory of tissue.

I must be pardoned, if indeed I ought not to be heartily thanked, for saying that I have long since learned, that no writers so clearly confess their ignorance of such points of histology, by *confounding* elements the very types of unlikeness, as those of the English publications I have named. With the single and very notable exception of Dr. Beale, no English writer's statements upon precisely such subjects, has any base but complete misunderstanding and ignorance of of the distinctive characters constituting the widest differences of the things he so glibly confounds or verbally pronounces to be alike. Emerson's opinion of Herbert Spencer, and his unsupported pretension to correctly appreciate and expound scientific facts, is far more pointedly applicable to the writer in question. The worst of these "stock" gentlemen is, they write without the slightest embarrassment from *facts*, and hence their statements are entitled to corresponding evidence.

It is abundantly obvious therefore, that the statement of this writer, as he phrazes it "of the connection between these extraordinary facts and the well known observation of Recklinghausen, on the presence of wandering contractile corpuscles in the plasmatic channels of the cornea, mesentery and connective tissue of other parts," is a mere embodiment of his ignorance and imagination. Since the contractile elements of Recklinghausen, were infered by him to be occasionally frequenters *normally* of these particular localities of tissue, and not at all as corpuscular constituents of the blood which issue upon and crowd into a scene of inflamation, from the blood vessels.

It is indeed a favorite design, of those who know not how otherwise to gain to the little,—to them childishly flattering notiriety,— by mention in scintific prints in connection with Virchow's famous doctrines, they so inordinately covet—to *attack* these doctrines. being always sure of abundant seconding therein in the English publications, simply because the writers of these publications are invariably "irritable" from that brawn and bell-born hatred (not brain born) of the sweeping truths especially of the famous discoverer in question, which disperse and clean away, old cumuli of error. Hence whenever a new pretender to the coveted honor of overturning his discoveries appears, these publications are officiously quick to introduce his claims, as giving the " death blow" to or as " subversive of" Virchows' discoveries. But what is stikingly remarkable is that no two of these dastroyers of Virchows cell doctrines, *establish any one particular in common*, but invariably antagonise each other in the particulars of their reputed discoveries. Thus for example, recently,[*Nov. No. Dental Cosmos.* Art. cell and other germinal forms] we pointed out one, who claimed to have " experimented" connective tissue itself into existence by blood-globules, " in which" as he phrazed it, " the blood is converted into connective tissue without formation of any kind of cells or nuclei," while in the instance in hand, we see another, who finds that these connective tissue corpuscles, have not the slightest morphological affiinity with the very same corpuscular elements of the blood, which latter are on the contrary *identical* with pus-corpuscles, therein demonstating as he supposes, that the connective tissue corpuscles, has nothing whatever to do with pus-corpuscles, they being identical with white blood corpucels. In short one finds, connective tissue to be in itself not an originally formed tissue, made up from elements intrinsic to it, like other tissues, but only and adventitious transformation of blood corpuscles escaped from their channels, and the other, while *denying* Virchows' doctrine that connective tissue is involved in the production of pus-corpuscels, (in which denial according to the English account consist his " death dealing" blow to this doctrine,) discovers the actual oneness and sameness of the white-blood and pus-corpuscles The latter body, therefore according to one, has no morphological connection with connective tissue corpusceles, but is identical with white blood corpuscels, while according to the other, the white blood corpuscles, gives rise to connective tissue. Thus one affirms that the blood corpuscles, identical according to the other with the pus-carpuscles, form connective tissue, while the latter denies that the latter tissue has anything to do with the pus-corpuscles identicai with the blood.

Yet according to English reporters, either of these gentlmen have destroyed Virchows' doctrine, by their wonderful discoveries. We venture to say, that if M. Conheim ever presented such claims as here represented before any "Berlin Soc." (what society?) unless he is of uncommonly stoney make up he came from this scene of his labors far better instructed, than when he entered.

(*To be Continued.*)

